# Magnetic Bead Manipulation in Microfluidic Chips for Biological Application

**DOI:** 10.34133/cbsystems.0023

**Published:** 2023-04-14

**Authors:** Gaozhe Cai, Zixin Yang, Yu-Cheng Chen, Yaru Huang, Lijuan Liang, Shilun Feng, Jianlong Zhao

**Affiliations:** ^1^ State Key Laboratory of Transducer Technology, Shanghai Institute of Microsystem and Information Technology, Chinese Academy of Sciences, Shanghai 200050, China.; ^2^ School of Communication and Information Engineering, Shanghai University, Shanghai 200444, China.; ^3^ School of Electrical and Electronics Engineering, Nanyang Technological University, 50 Nanyang Ave., Singapore 639798, Singapore.; ^4^ School of Life Sciences, Shanghai Normal University, Shanghai, 200235, China.; ^5^ Center of Materials Science and Optoelectronics Engineering, University of Chinese Academy of Sciences, Beijing 100049, China.; ^6^ Xiangfu Laboratory, Jiaxing, Zhejiang 314102, China.

## Abstract

Magnetic beads manipulation in microfluidic chips is a promising research field for biological application, especially in the detection of biological targets. In this review, we intend to present a thorough and in-depth overview of recent magnetic beads manipulation in microfluidic chips and its biological application. First, we introduce the mechanism of magnetic manipulation in microfluidic chip, including force analysis, particle properties, and surface modification. Then, we compare some existing methods of magnetic manipulation in microfluidic chip and list their biological application. Besides, the suggestions and outlook for future developments in the magnetic manipulation system are also discussed and summarized.

## Introduction

Microfluidics is widely regarded as one of the most promising and cutting-edge areas of research since the concept of Micro Total Analysis Systems was proposed by Manz et al. [1] in 1990. Microfluidics is a generalized term that describes the precise control and manipulation of small quantities of fluid behavior at the submillimeter scale. It can revolutionize the way in which various biological, chemical, and medical analyses are conducted, as it allows for the integration of a range of basic operations, such as sample preparation, reaction, separation, and detection, all within a microscale chip that can complete the whole analysis process in an automated fashion [2–4]. This approach has the benefits of requiring only minuscule fluid volumes (down to the nanoliter or even the picoliter range), which can greatly reduce analysis costs and reaction time, as well as being characterized by a large number of parallel processing and automated operation [5].

Concomitant with the rapid development of microfluidic systems, nanomaterials and nanoparticles have garnered increasing attention and become a focal point of recent researches. In particular, owing a large surface-to-volume ratio to enhance the interaction of reactive surfaces with passing fluids, magnetic beads have been demonstrated as a promising tool for capturing target analytes. Moreover, although there are many existing particle manipulation techniques in microfluidics, such as optical tweezers [6–8], acoustic manipulation [9], hydrodynamic manipulation [10,11], and dielectrophoresis [12], the manipulation of magnetic beads by magnetic fields has its unique advantages than those other methods, as it can be easily manipulated with permanent magnets or electromagnets. Besides, the control and manipulation of the magnetic beads in solution by magnetic field offer an effective technology for the transport and localization of materials toward biotargets [13]. As the control method for the magnetic beads is relied on external magnetic fields wirelessly without direct contact, it has, thus, been widely developed for applications such as magnetic drug and gene delivery [14,15], as well as magnetic separation [16] in microscale applications.

The magnetic manipulation combining with microfluidics has garnered considerable attention because of the fact that there are several meeting points between 2 technologies such as large surface-to-volume ratio and controllability. Microfluidics provides the microscale solution environment for magnetic beads, which further enlarge its surface-to-volume ratio to sample solution and reduce the reaction time. Moreover, microfluidic chip methods allow for greater flexible in the arrangement of external or internal magnets, which can lead more accurate and automated magnetic manipulation. As a result, the manipulation of magnetic beads in microfluidic chips has emerged as a promising research field for biological and chemical applications in recent years, and the progress in these areas has been reviewed by numerous high-quality publications [17–19].

**Fig. 1.**
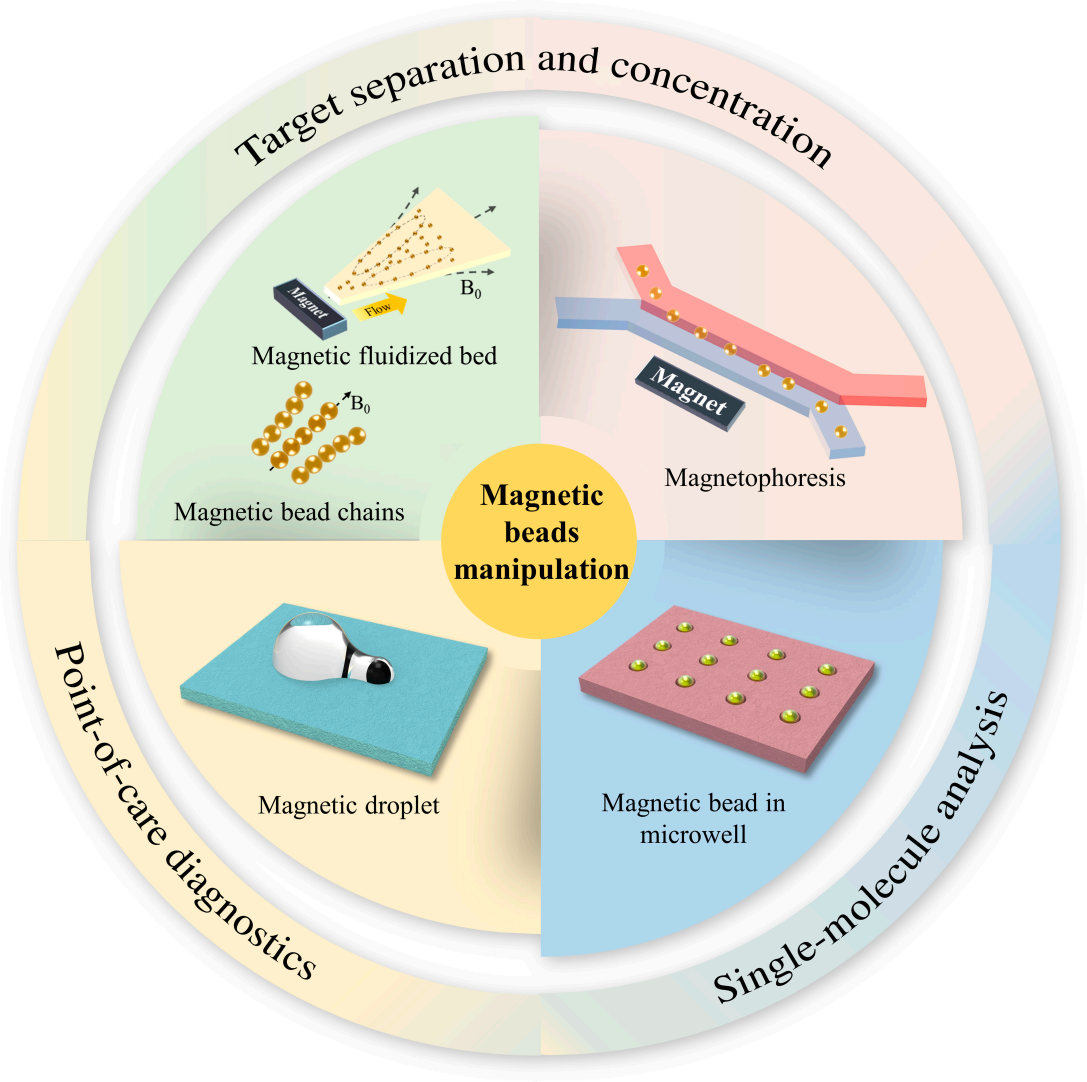
An overview of magnetic beads manipulation method in microfluidics and its biological applications.

In this review, our aim is to present a thorough and in-depth overview of recent magnetic bead manipulation in microfluidic chip and its biological application. We will begin by presenting a detailed introduction to the fundamental concepts of superparamagnetism, followed by a comprehensive calculation and discussion of the various forces that come into play when magnetic beads are subjected to a microfluidic and magnetic environment. Meanwhile, the mechanism of the forming of magnetic bead chains is also introduced on the basis of the force calculation. Then, we summarize recent magnetic bead manipulation methods according to the formation and state of the magnetic beads such as magnetophoresis, magnetic bead chains, and magnetic fluidized bed (Fig. 1). Finally, the biological applications of each magnetic bead manipulation methods in microfluidics are listed. The conclusion highlights some suggestions and outlook for future developments in the magnetic manipulation system.

## Basic Knowledge of Magnetic Manipulation in Microfluidic Chip

### Magnetic beads and surface modification

Magnetic beads in biological application are generally synthesized using coprecipitation method [20] and composed of a magnetic core, a surface coating, and specific biological recognition element situated on the surface. The magnetic core is a critical component of the magnetic beads, providing the necessary magnetic properties for their magnetic manipulation within varying magnetic fields. To date, the materials utilized in the magnetic core predominantly consist of pure metals (e.g., Co, Fe, and Ni), their respective oxides, or transition-metal-doped oxides and metal alloys (e.g., CoPt_3_, FeCo, and FePt). Of these materials, iron oxides are considered to be the most widely utilized materials for biological applications due to their favorable characteristics, such as excellent magnetic property, controllable morphology, low cost, and good biocompatibility [21].

The magnetic beads synthesized without a protective coating possess hydrophobic surfaces that can lead the agglomeration of the magnetic beads and, thus, hinder surface immobilization with biological recognition elements [22]. To protect these magnetic beads from both oxidation and agglomeration, the surface coating and functionalization procedure should be performed using nonpolymeric stabilizers based on organic monomers or polymeric stabilizers [23].

Following surface modification with a variety of reactive functional groups such as amines and carboxyls, the magnetic beads can be coupled with specific biological recognition elements such as antibodies, aptamers, peptides, etc. according to required applications. Antibodies are commonly used for their high specificity in recognizing target antigens, and there are 3 main methods for modifying antibodies onto magnetic beads: (a) direct covalent linkage between the carboxyl and amino groups through ethyl(dimethylaminopropyl)carbodiimide/*N*-hydroxysuccinimide chemistry, which is characterized by its low cost and short reaction time [24]; (b) indirect coupling via specific antibody binding proteins such as protein A or protein G [25]; and (c) indirect avidin–biotin binding between the streptavidin or avidin on the beads and the biotinylated antibodies [26].

As another important biological recognition element, aptamers are widely used in analytical applications. Aptamers are synthetic single-stranded DNA or RNA molecules selected in vitro using a process called systematic evolution of ligands by exponential enrichment, which involves iterative rounds of selection and amplification. The modification strategies of aptamer on magnetic beads are similar to antibodies. One is to introduce amine groups on one terminus of the aptamer for direct covalent linkage with carboxylated beads by ethyl(dimethylaminopropyl)carbodiimide/*N*-hydroxysuccinimide method. This method allows for the oriented immobilization of aptamers, which can offer the advantage of enabling specific modifications to be attached to particular positions within the aptamer sequence. Streptavidin-coated magnetic beads can also be used to connect with aptamers labeled with biotin [27].

### Force analysis

The magnetic bead possesses the capability of becoming magnetized through the generation of a magnetic moment (***M***_**b**_) when a magnetic field (***H***) is applied. The magnetic moment aligns with the direction of the magnetic field and can be calculated as below [28,29]:

$$M_{b}=\chi_{m}H=\frac{3\chi_{b}}{\chi_{b}+3}H$$ (1)

where *χ*_m_ represents the magnetic susceptibility. In addition, the susceptibility of a magnetic bead *χ*_b_ is defined as: *χ*_b_ = *μ*_b_ / *μ*_0_ − 1, where *μ*_b_ represents the relative permeability of the magnetic beads and *μ*_0_ is the permeability in vacuum, given by 𝜇_0_ = 4𝜋 × 10^−7^ 𝑁/𝐴^−2^. Furthermore, when the magnetic bead is placed in a medium of susceptibility (*χ*_f_), Eq. 1 can be rewritten as [30]:

$$M_{b}=\frac{3\chi_{b}\left(\chi_{f}+1\right)}{\left(\chi_{b}-\chi_{f}\right)+3\left(\chi_{f}+1\right)}H$$ (2)

An equivalent dipolar magnetic moment ***m***_**b**_ is usually used to determine the magnetic force acting on the magnetic bead using the following equation [30]:

$$m_{b}=\frac{3V_{b}\left(\chi_{b}-\chi_{f}\right)}{\left(\chi_{b}-\chi_{f}\right)+3\left(\chi_{f}+1\right)}H$$ (3)

When facing microscale, the magnetic force supporting a magnetic bead movement is primarily driven by the interplay between the interaction energy of the magnetic bead and the magnetic field (*E^H^*), as well as the interaction energy between 2 beads (*E^m^*) [31]:

$$E^{H}=-m_{b}\cdot B$$ (4)

$$E_{\mathrm{ij}}^{m}=\frac{\mu_{0}m_{i}m_{j}}{4\pi {r_{\mathrm{ij}}}^{3}}\left\{n_{i}\cdot n_{j}\cdot 3\left(n_{i}\cdot t_{\mathrm{ij}}\right)\left(n_{j}\cdot t_{\mathrm{ij}}\right)\right\}$$ (5)

where *r_ij_* is the modulus of the ***r***_***ij***_ and ***n***_***i***_ and ***t***_***ij***_ are the unit vector of ***m***_***i***_ and ***r***_***ij***_, respectively. Using the Eq. 4, the interaction magnetic force between bead and magnetic field (***F***_**mb**_) can be expressed as:

$$F_{\mathrm{mb}}=\cdot ▽\left(E^{H}\right)=▽\left(m_{b}\cdot B\right)$$

$$\begin{array}{c} =\left(m_{b}\cdot ▽\right)B+\left(B\cdot ▽\right)m_{b}+m_{b}\times \left(▽\times B\right)+B\times \left(▽\times m_{b}\right) \end{array}$$ (6)

The third and fourth terms in Eq. 6 can be ignored because of the Ampere’s law, as there are no currents induced in the magnetic beads. Besides, the second term in Eq. 6 can also be vanished when the dipole moment is constant in space. Using the relationship ***B*** =*μ**H*** and Eq. 3, ***F***_**mb**_ can be rewritten as:

$$F_{\mathrm{mb}}=\frac{3\mu_{f}V_{b}\left(\chi_{b}-\chi_{f}\right)}{\left(\chi_{b}-\chi_{f}\right)+3\left(\chi_{f}+1\right)}\left(H\cdot ▽\right)H$$ (7)

where ***F***_**mb**_ depends on the volume of the beads (*V*_b_), the difference in the magnetic susceptibilities between beads and the suspending medium (*χ*_b_ − *χ*_f_), and the magnitude and gradient of the applied magnetic field (***H***) [32]. The aforementioned equation implies that a marked magnetic force can only be realized when both the magnetic field and its gradient attain magnitudes of notable values. Besides, when in a paramagnetic solution (where *χ*_f_ > 0), a diamagnetic bead (*χ*_b_ < 0) is subjected to a force in the direction of the magnetic field gradient. Conversely, when in a diamagnetic solution (where *χ*_f_ < 0), a paramagnetic bead (*χ*_b_ > 0) experiences a force in the direction of the magnetic source [33].

When the magnetic bead is in a fluid, in addition to the magnetic force, the magnetic bead is also subjected to a drag force (***F***_**d**_) of the fluid. Under the condition of microflow, the Reynolds number is usually much smaller than one belonging to the laminar flow. According to Stokes’ law for low Reynolds number, the drag force exerted on particles can be calculated using following equation:

$$F_{d}=6\mathrm{\pi \eta r}\left(V_{f}-V_{p}\right)f_{D}$$ (8)

where *η* is the viscosity of the surrounding medium, *r* is the radius of magnetic bead, ***V***_**f**_ is the flow rate of fluid, ***V***_p_ is the one for particle, and *f*_D_ is the hydrodynamic drag coefficient.

### Magnetic bead chain mechanism

When multiple magnetic beads are magnetized under the action of magnetic field, there will be an interaction magnetic force between bead and bead (***F***_**bb**_), which can be calculated using Eqs. 3 and 4:

$$\begin{array}{c} \begin{array}{c} F_{\mathrm{bb}}=-▽\left(E_{\mathrm{ij}}^{m}\right)=\\ -▽\left\{\frac{\mu_{0}m_{i}m_{j}}{4\pi {r_{\mathrm{ij}}}^{3}}\left[n_{i}\cdot n_{j}-3\left(n_{i}\cdot t_{\mathrm{ij}}\right)\left(n_{j}\cdot t_{\mathrm{ij}}\right)\right]\right\} \end{array} \end{array}$$ (9)

Equation 9 can be replaced by the following equation when the adjacent magnetic beads have the same volume:

$$F_{\mathrm{bb}}=-\frac{\mu_{0}m_{i}m_{j}}{4\pi }▽\left(\frac{1-3\;{\text{cos}}^{2}\theta }{{r_{\mathrm{ij}}}^{3}}\right)$$ (10)

where *θ* represents the angle between the center linking line of the 2 magnetic beads and the direction of the magnetic field. The components of ***F***_**bb**_ in ***r*** and ***θ*** can be expressed as:

$$F_{r}=-\frac{\mu_{0}m_{i}m_{j}}{4\pi }\frac{\partial \left(\frac{1-3\;{\text{cos}}^{2}\theta }{{r_{\mathrm{ij}}}^{3}}\right)}{\partial r_{\mathrm{ij}}}=\frac{3\mu_{0}m_{i}m_{j}\left(1-3\;{\text{cos}}^{2}\theta \right)}{4\pi {r_{\mathrm{ij}}}^{4}}$$ (11)

$$F_{\theta }=-\frac{\mu_{0}m_{i}m_{j}}{4\pi r_{\mathrm{ij}}}\frac{\partial \left(\frac{1-3\;{\text{cos}}^{2}\theta }{{r_{\mathrm{ij}}}^{3}}\right)}{\partial \theta }=\frac{6\mu_{0}m_{i}m_{j}\text{sin}\theta \text{cos}\theta }{4\pi {r_{\mathrm{ij}}}^{4}}$$ (12)

When the *θ* is smaller than 54.73°, we can find that ***F***_***r***_ is calculated to negative, which means that the 2 magnetic beads will be attracted to each other. The force ***F***_***θ***_ is positive in the whole line, resulting in the bead moves in a counterclockwise direction [19]. Therefore, the chainlike clusters are formed, aligned to the applied magnetic field direction, and they become thickly clustered with an increase in the strength of the magnetic field [34,35]. Note that the magnetic force ***F***_**mb**_ can be generated only when the magnetic field gradient exists, resulting in the translation of beads, while the aggregation of beads only appears under the uniform magnetic field.

## Magnetic Bead Manipulation in Microfluidic Chip

### Magnetic beads in microfluidics

Combining magnetic beads with microfluidic devices offers several advantages for a wide range of applications, such as biomolecular analysis, biosensing, and drug discovery. The combination of these 2 technologies allows for precise and rapid manipulation of magnetic beads in microfluidic channels, leading to enhanced sensitivity, speed, and specificity in many assays. One of the main advantages is the ability to perform multiplexed assays, where multiple targets can be simultaneously detected in a single experiment. The integration of magnetic beads and microfluidics allows for high-throughput and parallel processing of multiple samples, leading to increased efficiency and reduced costs. Another advantage of this combination is the ability to selectively capture and isolate targets from complex samples. Magnetic beads functionalized with specific biological recognition elements can be used to capture and enrich target molecules from a sample, while nonspecific molecules can be washed away. This selective capture and enrichment can improve the sensitivity and specificity of many assays in microfluidic chip.

However, there are several complications that need to be addressed when combining magnetic beads with microfluidic devices. One major issue is the potential for clogging of the microfluidic channels by the magnetic beads or the self- agglomeration of the magnetic beads. To prevent this, proper channel and magnetic field design and control of the magnetic forces are essential. Besides, the surface properties of the microfluidic channel can also affect the adherence of particles to the surface. Surface modifications can be used to control the surface wettability and reduce nonspecific adsorption of particles, which can help to reduce clogging and improve the efficiency of the assay. In addition, the use of magnetic beads in microfluidic devices can interfere with some detection methods, such as fluorescence, due to light scattering. To overcome this, alternative detection methods, such as electrochemical or directly magnetic detection, can be used. In the following sections, we will provide a detailed overview of magnetic bead manipulation techniques in microfluidics and how to address the above complications while achieving their biological applications.

### Magnetophoresis

**Fig. 2.**
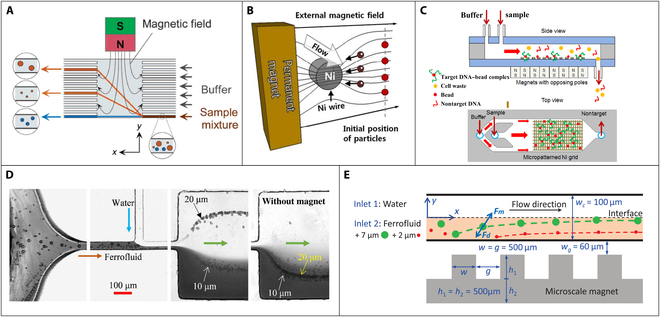
(A) The principle of on-chip system using free-flow magnetophoresis for separation magnetic beads with different sizes. Reproduced with permission from Pamme and Manz [37]. Copyright 2004, American Chemical Society. (B) Principle of the magnetophoresis using a ferromagnetic nickel wire. Reproduced with permission from Nam et al. [40]. Copyright 2013, American Chemical Society. (C) The target DNA–bead complexes are absorbed on the edge of the nickel grid, while the nontarget DNA flows directly out of the exit. Reproduced with permission from Hale and Darabi [44]. Copyright 2014, American Institute of Physics. (D) Picture of continuous separation of 20-μm nonmagnetic particles from 10-μm ones in a T-shaped microchannel. Reproduced with permission from Chen et al. [50]. Copyright 2017, American Chemical Society. (E) Principle of the focusing and separating diamagnetic beads using 2 coflowing fluids. Reproduced with permission from Zhou and Wang [51]. Copyright 2016, AIP Publishing.

Magnetophoresis is a conventional continuous magnetic separation method, which was first reported by Degen et al. [36] in 1977. The targets in the sample are first reacted and combined with enough magnetic nanobeads to form bead–target complex (magnetic target). The sample flow is then introduced into a magnetophoresis channel along with a buffer solution, and a gradient magnetic field is applied. The magnetic targets experience a force in the direction of the magnetic field gradient, causing them to migrate toward the higher gradient direction. Consequently, the magnetic targets are isolated continuously from the nonmagnetic sample background, resulting in a continuous flow separation. Finally, the magnetic targets are collected at the designed outlet. In the same magnetic field and suspending medium, 2 key parameters that importantly influence the magnetophoresis process are the size of the beads and their magnetic susceptibility. Pamme and Manz [37] demonstrated an on-chip system of free-flow magnetophoresis as shown in Fig. 2A. With the change of magnetic susceptibility, size, and flow velocity, the magnetic beads are deviated from the direction of laminar flow under a perpendicular magnetic field up to 500 mT. Results showed that 2 sizes of superparamagnetic beads with different magnetic susceptibilities could be separated from each other in the channel at a flow rate of 0.3 mm/s. Wang et al. [38] proposed a 3-dimensional (3D) printed magnetophoresis system that was first used for H5N1 virus separation. The virus targeted with magnetic nanobeads was deviated into the bottom channel under a magnetic field gradient with a separation efficiency up to 88%.

One important factor in magnetophoresis is the magnetic force exerted by the external magnetic field [39]. Although using the permanent magnets could easily generate magnetic field without another external power supply, the resulting magnetic field is prone to attenuation. This can lead to an inhomogeneous magnetic force that may impact magnetic separation processes. Using micropermanent magnets integrated in the microfluidic chip could overcome the attenuation. However, it may not be suitable for high-flow scenarios due to the limited magnetic force generated. One solution is to use soft ferromagnetic microstructure, such as nickel (e.g., ferromagnetic nickel wire and nickel microarray), and subject it to the external magnetic field (Fig. 2B) [40–43]. By integrating ferromagnetic microstructure, the magnetic field gradient in its vicinity can be increased to generate a large magnetic force for magnetic separation. For instance, Hale and Darabi [44] developed a microfluidic chip for rapid isolation of DNA from human blood using a positive selection method based on magnetophoresis (Fig. 2C). In this design, a patterned thin nickel grid is deposited on a glass substrate to generate a high gradient magnetic field array for magnetic separation. The results indicated that this proposed method can separate and purify up to 33 μg of DNA in 1 ml of blood sample with a high flow rate of 20 ml/h. The electromagnets can also be used for magnetophoresis, as it can be easily controlled and integrated into microfluidic chip [45–47]. Chung et al. [45] proposed a particle sorting chip integrated with microelectromagnet and cooling channel to reduce Joule heat generated by electromagnets. The results showed that this proposed microfluidic chip owned a high separation efficiency (85%) when the current was 1A.

Another key factor in magnetophoresis is the hydrodynamic force resulting from the flow of fluids [39]. Some special microchannel structures were designed to produce high separation efficiencies. Although the most common one is the straight microchannel due to its simplicity, alternative structures, such as serpentine and L-, T-, and U-shaped channels, have also been proposed. Wu et al. [48] developed a novel design of the microfluidic chip for continuous magnetophoretic separation using L/T-shaped channels. The L/T-shaped structure could induce a special flow field to enhance the separation efficiency. The results of the study indicated that the separation efficiencies of L- and T-shaped microchannel separators were 63.4% and 100%, respectively, compared to just 43.7% for the straight microchannel. In a similar study, Han et al. [49] proposed a novel method for improving the separation efficiency of magnetophoresis by introducing a broadened segment in the T-shaped microchannel and controlling the flow rate ratio of 2 fluid streams. Besides, some undesirable impurities in the magnetic bead solution may also affect the separation efficiency. It is crucial to effectively remove the solvents and some undesirable impurities in the magnetic bead solution before magnetic separation. Chen et al. [50] designed a microfluidic system for continuous separation and washing of nonmagnetic beads using a simple T-shaped microchannel (Fig. 2D). The large particles were separated by the combined effect of magnetic force and inertial lift in the flows, while the small particles remain in the ferrofluid due to insufficient magnetic force; notably, the outlet position of particles varies with the flow rate.

In magnetophoresis, the accumulation of magnetic nanobeads on the side wall of the microchannel poses a major problem. With prolonged operation, the nanoparticles can block the channel. In addition, a minor portion of impurities in the sample stream may follow the target flow, leading to incomplete separation. To solve these limitations, Zhou and Wang [51] proposed a novel approach for separating diamagnetic microparticles utilizing a laminar fluid interface induced by 2 coflowing fluids in a straight channel (Fig. 2E). The strong force generated by the induced magnetic field deflects and focuses large particles at the interface, while the small particles keep their initial trajectory.

Compared to the conventional magnetic separation method in tube, magnetophoresis offers a continuous and automatic approach to separating targets from a theoretically unlimited volume of solution, which has a great significance for large volume sample pretreatment. The magnetic bead-conjugated target can be efficiently separated from complex samples due to the high gradient magnetic field. However, as the magnetic beads always flow together with the sample, a large amount of immune magnetic beads is often required to ensure a high separation efficiency for large volume samples, leading to a high cost.

### Magnetic bead chains

Fixing the immune magnetic nanobeads in a certain area of microfluidic channel is an ideal solution to reduce the excessive use for immune reaction and separation. However, there are 2 major challenging issues for fixing magnetic beads in the channel. One is the agglomeration of the magnetic beads on the inner surface of the channel when the magnet is placed close to channel, which will reduce the effective area of beads exposed to the solution and the capture efficiency. By placing the magnet away from the channel with an appropriate distance, the magnetic beads can be evenly distributed. Then, the other challenge is coming that is how to keep the stability of the magnetic beads in the channel as the magnetic force is not enough to remain them under the fluidic flow with high rate. To overcome these issues, efforts have been made to create the stable magnetic bead chains in microfluidic chip with different magnetic fields. As mentioned and analyzed in the Magnetic bead chain mechanism section, the magnetic beads can be self-assembled into chains due to dipole-to-dipole interaction under a special magnetic field [52]. The formed bead chains can be either fixed in the microfluidic channel or remained freely in an area. Therefore, the magnetic bead chain methods could be divided into dynamic and static one.

#### Static magnetic bead chains

**Fig. 3.**
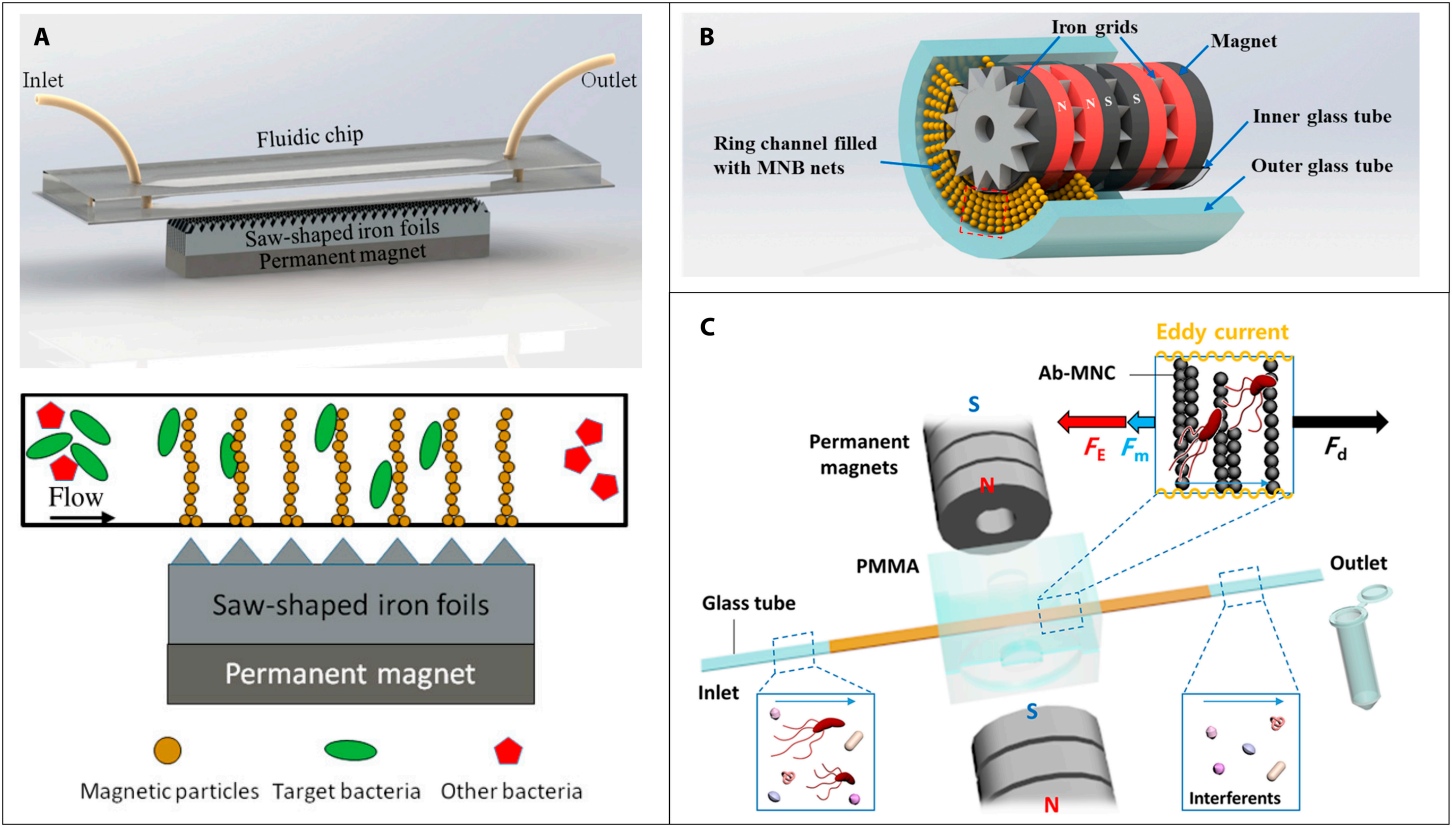
(A) Principle of the static magnetic bead chains separation using dot-array magnetic field with high gradient. Reproduced with permission from Cai et al. [53]. Copyright 2018, Multidisciplinary Digital Publishing Institute. (B) Principle of the static magnetic bead chains separation using repelling magnets and iron grids inside coaxial glass channels. MNB, magnetic nanobead. Reproduced with permission from Xue et al. [56]. Copyright 2021, Elsevier. (C) Principle of the static magnetic bead chains separation using a perpendicular magnetic field and copper-tape-wrapped glass channel. PMMA, polymethyl methacrylate; Ab-MNC, antibody-immobilized magnetic nanobead chains. Reproduced with permission from Lee et al. [57]. Copyright 2019, American Chemical Society.

High gradient magnetic field was usually used to create static magnetic chains in microfluidic channel. Cai et al. [53] proposed a dot-array magnetic field with high gradient by laminating sawtooth-shaped iron foils. As shown in Fig. 3A, the magnetic field could be focused at the tip of foils, and the formed bead chain array were distributed across the channel, thereby increasing the effective collision between target bacteria and magnetic beads. This magnetic separation method was able to separate 80% of target bacteria from 500 μl of sample. To form higher chains in the microfluidic channel for the separation of bacteria in large volume sample, some studies based on coaxial capillary and magnetic grid field were proposed by Lin’s group [54–56]. As shown in Fig. 3B, the basic principle of these studies was utilizing adjacent repelling magnets to magnetize iron balls or grids inside coaxial glass channels to generate local high gradient magnetic fields near the surface of the channel to form magnetic bead chains. A magnetic separation efficiency of 60% for *Salmonella* in 10 ml of sample was achieved by Xue et al. [56], and a lower detection limit of 19 colony-forming units (CFU)/ml within 1.5 h was realized by combining with manganese dioxide nanoflowers for signal output and an interdigitated microelectrode for signal detection.

To make the static bead chains more stable to resist higher flow rates, Lee et al. [57] presented a magnetic nanobead chain virtual net method by placing 2 permanent magnets on both sides of a glass channel. As shown in Fig. 3C, the glass channel was wrapped with a copper tape, which can provide a magnetic force in an opposite direction to the flow when the magnetic beads were pushed to the conductive region under an external magnetic field based on the Lenz’s law. The opposite magnetic force can increase the stability of the formed magnetic nanobead chain in the channel at high flow rate. Results showed that even if the flow rate increased to 1 ml/min, this proposed method could still separate target bacteria with an efficiency of 85%.

Static magnetic bead chains offer the advantage such as high integration and simple operation and could realize continuous flow capture and separation of target from complex samples. Hence, it is more suitable for food safety field, as the volume of food sample is large and the sample matrix is usually complex.

#### Dynamic magnetic bead chains

**Fig. 4.**
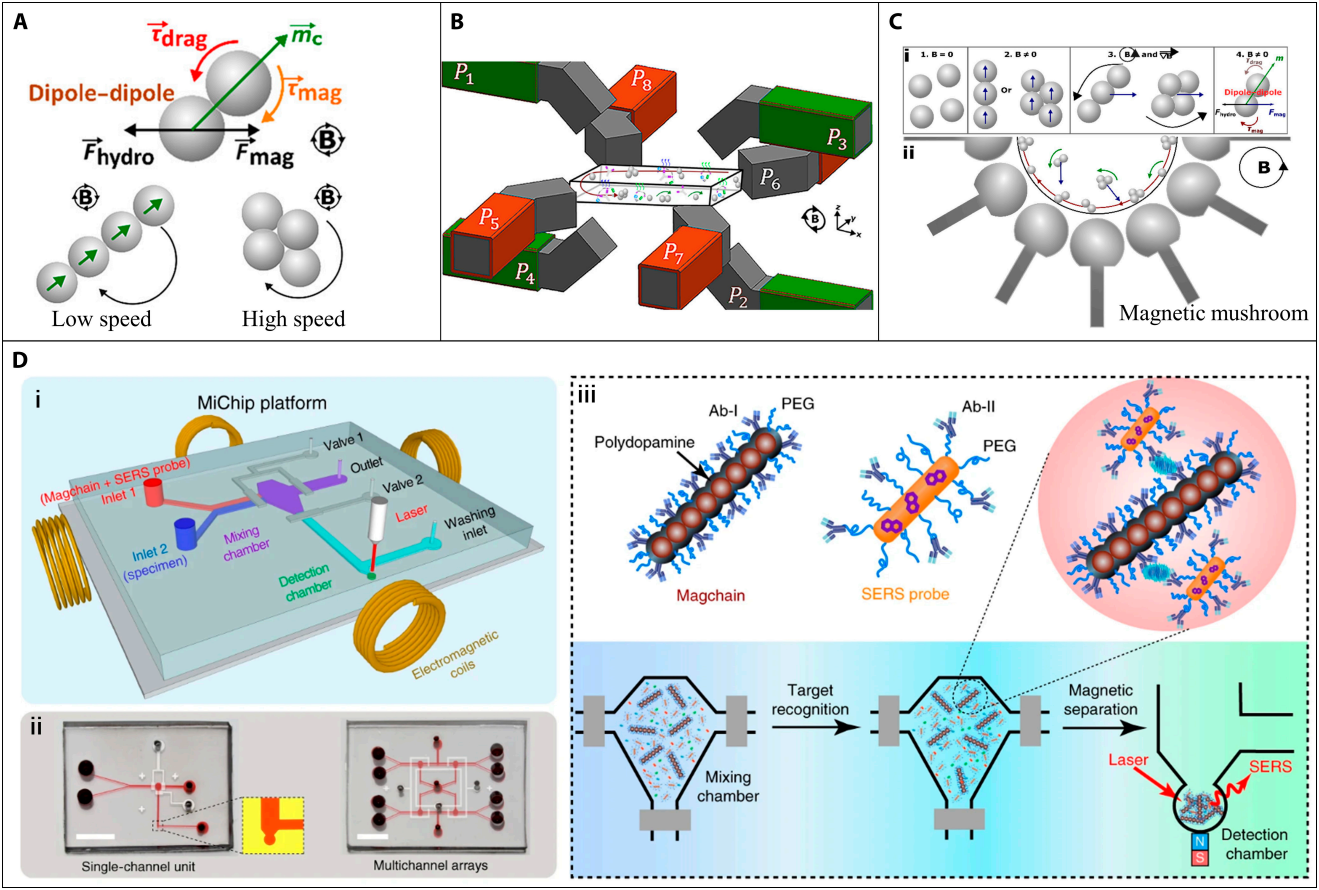
(A) Dynamic behavior of magnetic beads in microfluidic channels within the rotating magnetic field. (B) Electromagnetic setup for the rotating magnetic field. Reproduced with permission from Shanko et al. [62]. Copyright 2021, American Chemical Society. (C) Working principle of the in-plane counterclockwise rotating external magnetic field. Reproduced with permission from Shanko et al. [63]. Copyright 2022, Springer. (D) (i) Schematic illustration of the Magchain-integrated microchip (MiChip) assay platform. (ii) Photographs of the MiChip. (iii) The principle of the MiChip assay for the detection of biomarkers. PEG, polyethylene glycol. SERS, surface-enhanced Raman scattering. Reproduced with permission from Xiong et al. [65]. Copyright 2018, Springer Nature.

The dynamic behavior of magnetic bead chains in microfluidic channels can be achieved by applying a rotating external magnetic field. This phenomenon is determined by the phase lag of the bead chains, which is an important parameter that influences the alignment and stability of the bead chains. It can be defined as the angle between the long axis of the magnetic bead chain and the externally applied magnetic field ***B*** (as shown in Fig. 4A) [58]. It can result in the deformation or fragmentation of the chain when the rotating speed of the external field increases beyond a certain point. This phenomenon is due to the repulsive nature of the magnetic dipole–dipole interaction that is responsible for chain formation. At slow rotation speeds, the bead chains can rotate with the field change, but at higher speeds, the repulsive interaction becomes dominant and can cause chain deformation or breakage, as depicted in Fig. 4A. This mechanism can be widely applied to realize fluid mixing [59,60] and magnetic separation [61] using dynamic assemble magnetic bead chains as active micromixers. Shanko et al. [62] presented a novel magnetic actuation configuration capable of inducing the phenomenon of “magnetic particle swarming” (MPS) in a microfluidic chamber, which resembled bird swarming. In contrast to conventional methods, MPS involved not only local rotational motions of magnetic bead chains but also a global rotational motion throughout the microchamber, which could well improve the micromixing for antibody-based assay. A custom-built electromagnetic setup was used to generate a rotating magnetic field, as depicted in Fig. 4B. Four electromagnets (*P*_1_ to *P*_4_) and 2 pairs of electromagnets (*P*_5_ to *P*_8_) in the horizontal plane were combined together to generate a rotating magnetic field and enable 3D manipulation of the magnetic beads. The results revealed that the MPS could only be observed at a magnetic field strength of at least 20 mT and frequencies between 10 and 60 Hz. To improve the visual detection of the process occurring in the microfluidic chip, magnetically mushroom-shaped structures (Fig. 4C) were integrated around the chamber in the above magnetic system to generate in-plane rotating magnetic field [63].

Preassembling the magnetic bead chains and locking the structure could resist the viscous drag torque and keep the chain structure complete under rapid rotating magnetic field. Xiong et al. [64,65] used ploydopamine to cross-link magnetic nanobeads to form nanochains with a width of 400 nm and a length of 20 μm, which functioned as nanoscale stir bars for rapid liquid mixing and as capturing agents for specific bioseparation, as depicted in Fig. 4D. The ploydopamine can be served as a scaffold to maintain the nanochains structure, and its surface functionalization allowed the immobilization of specific biological recognition elements. Results showed that the nanochains exhibited a maximum mixing efficiency of about 80% within 60 s, rotating at 540 rpm. Furthermore, in this study, the combination of nanochains and surface-enhanced Raman scattering technology enabled the quantification of a panel of cancer protein biomarkers [prostate-specific antigen (PSA), α-fetoprotein (AFP), carcinoembryonic antigen (CEA), carbohydrate antigen 125, and carbohydrate antigen 199] and bacterial species (*Escherichia coli* O157:H7) in 1 μl of body fluids within 8 min.

### Magnetic fluidized bed

**Fig. 5.**
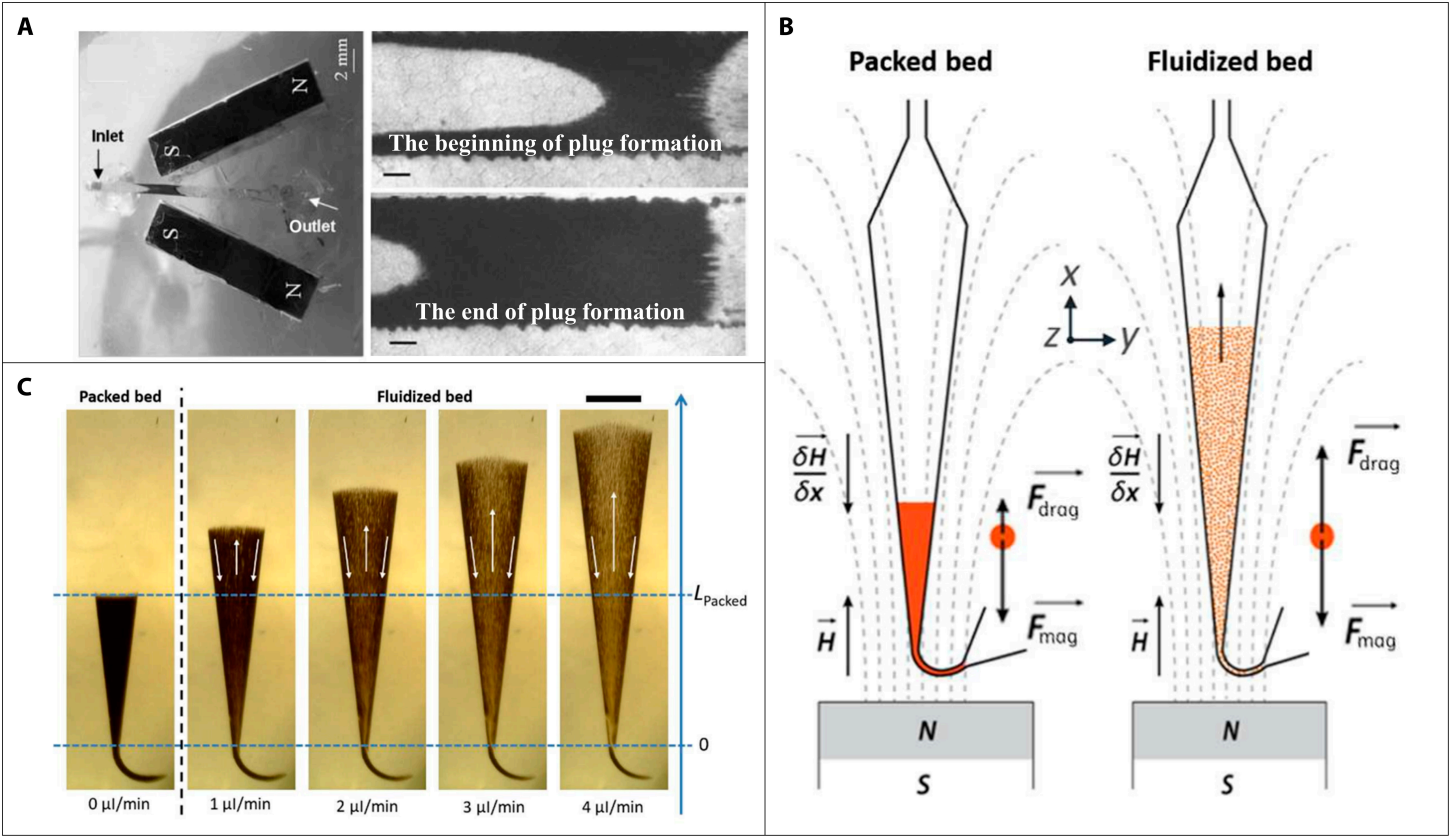
(A) Picture of the magnetic bead chain chip with a single pair of magnets and the magnetic beads plug. Scale bars, 200 mm. Reproduced with permission from Le Nel et al. [67]. Copyright 2008, Royal Society of Chemistry. (B) Principle of the microfluidic fluidized bed at different working regimes. (C) Images of the fluidized bed under different flow rates. Scale bar, 1 mm. Reproduced with permission from Pereiro et al. [68]. Copyright 2017, Royal Society of Chemistry.

With the same purpose of dynamic magnetic bead chains, magnetic fluidized bed is a method that could also keep the magnetic bead dynamic moving in a fixed area, which is generated when the viscous drag and magnetic force acted on the magnetic beads are in dynamic stable state and served as counterbalancing force. Tabnaoui [66] conducted the static and dynamic magnetic bead plug research based on the magnetic bead chain chip (Fig. 5A) with a single pair of magnets [67] and proposed the concept of magnetic fluidized bed for the first time. Two magnets were placed on both sides of the microfluidic chip to generate a magnetic trap region across the microchannel to against the fluidic drag force. At optimized flow rate, the magnetic fluidized bed was formed by the dynamic balance between drag force and magnetic force. However, the formed magnetic bead plug was not homogeneous, and an obvious fracture was observed under the designed magnetic field, which may affect the kinetics of fluid–solid interactions.

To solve this limitation, Pereiro et al. [68,69] optimized the magnetic field and the shape of the channel and proposed a new design of the microfluidic device (Fig. 5B) for generating magnetic fluidized bed. The introduction of a permanent magnet in the entrance of a V-shape microfluidic chamber induced a magnetic field gradient collinear with the fluid flow. As shown in Fig. 5C, the magnetic beads remain statically packed near the entrance of the chamber when a low pressure was applied. However, when the input pressure exceeded a threshold value, the magnetic beads experienced an acceleration force and were pushed downstream toward a specific position by the strong drag force near the entrance of the chamber, owing to the narrow channel. At that position, the magnetic and drag forces balanced each other, causing the magnetic beads to continuously displace toward the sides of the chamber due to the collision with other beads. As a result of the local nonslipping condition near the chamber wall, the drag forces weaken, and the magnetic forces became dominant, returning the beads to the entrance, resulting in a magnetic fluidized bed. This fluidized bed exhibited a constant recirculation of magnetic beads with almost uniform density in the microfluidic channel, providing efficient bead–liquid mixing for immunocapture of various biotargets [70–72]. Hernández-Neuta et al. [71] presented a magnetic-fluidized-bed-based method to realize an integrated DNA assay. In their work, except for placing permanent magnet in front of the entrance, 2 similar experiments using rotating and static permanent magnets placed on the top of the microfluidic chip were conducted for comparison. The findings of this study revealed that the magnetic fluidized bed method exhibited greater efficiency compared to the other 2 modes, highlighting that the dynamic regime of the fluidized bed could create an optimal condition for achieving rapid hybridization kinetics.

As described on the above sections, these 3 methods (magnetophoresis, magnetic bead chains, and magnetic fluidized bed) are commonly used for biotarget separation and enrichment in the microfluidic chip. The key point of these methods is the control of the movement or static state of the magnetic beads in the microfluidic channel through the magnetic field to ensure that the magnetic beads can stay the largest effective specific surface area to improve the reaction with the biotarget. In the Magnetic droplet and Magnetic bead in microwell sections, we will introduce some other magnetic bead manipulation methods to achieve automatic biological detection and point-of-care testing in the microfluidic chip.

### Magnetic droplet

In the realm of microfluidics, digital microfluidics (DMF) has emerged as an active research field for the past 10 years. This technology is being increasingly utilized in point-of-care testing, where complex sample preparation is necessary. DMF could manipulate nanoliter to microliter droplets on a plain surface. Depending on the actuation mechanism used, DMF can be classified into various categories, including electrowetting on dielectric [73–75], magnetic [76–78], surface acoustic wave [79,80], and other types [81,82]. Despite the widespread popularity of the electrowetting-on-dielectric actuation method, the magnetic actuation approach continues to offer important and irreplaceable benefits, as noted by Zhang and Nguyen [83]. Magnetic DMF could manipulate the magnetic-bead-contained droplets by controlling permanent magnets, electromagnets, or hybrid magnetic fields. The movement of the magnetic droplets on a plain surface was mainly determined by the magnetic force and interface resistance. Therefore, substrate with low surface tension and adhesion is ideal for the magnetic droplet manipulation. Teflon amorphous fluoropolymer is a standard hydrophobic surface coating material used for DMF devices [78]. It can provide a hydrophobic and oleophobic substrate for droplet movement. Under selective modification on different areas of the substrate, various operations on the droplets can be realized, such as splitting, moving, and mixing. Besides, some natural substrate was also used as the substrate in magnetic DMF. Mats et al. [84] found that Colocasia leaf owned a contact angel of over 150°, and the magnetic manipulation was successfully achieved on that surface.

The control of the balance between magnetic force and interface resistance is critical and challenging in magnetic droplet manipulation. Long et al. [85] demonstrated the fundamentals of the magnetic droplet operation. Their work also explained the influence of magnetic, friction, and capillary-induced drag forces on magnetic droplet movement and the effects of particle type, droplet size, surrounding oil layer, surface tension, and viscosity. The magnetic force is affected by multiple parameters such as the magnetism of the magnetic bead itself, the amounts and volume of the magnetic beads, as well as the intensity and the moving speed of the external magnetic field. In many biological application scenarios, the conditions of the magnetic bead itself are not easy to be changed, so adjusting the magnetic field is the most effective way to control the magnetic force.

#### Droplet manipulation using permanent magnets

Moving the permanent magnets is one common magnetic field control method, and numerous studies have showcased the application of the manipulation of droplets using permanent magnets for bioanalytical assays [86–88]. Huang et al. [89] proposed a droplet-array-based microfluidic system to perform chemiluminescence immunoassay programmatically. The antibody-conjugated magnetic beads in the droplet were used for capturing target, and the alkaline-phosphatase-labeled antibodies were used for detection. By moving the permanent magnet, the magnetic beads can be gathered, released, transferred, and mixed between droplets. In addition, combined with the optical detection system, procalcitonin with a concentration of 0.044 ng/ml could be detected within 12 min. To improve the analysis speed and reduce the cross-contamination in conventional 2D droplet platform, Park et al. [90] proposed a 3D magnetic manipulation strategy. The reagent droplet containing magnetic beads could be transported vertically by a permanent magnet on the top and merged with another reagent droplet. By enlarging the droplet operation system into a 3D space, the microfluidic density and analysis speed were increased.

**Fig. 6.**
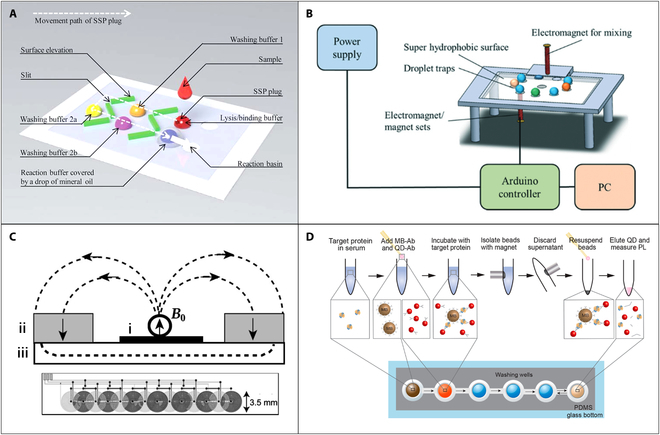
(A) Schematic illustration of the microfluidic chip used surface topographic feature to facilitate the droplet manipulation. SSP, silica superparamagnetic particles. Reproduced with permission from Zhang et al. [76]. Copyright 2010, Royal Society of Chemistry. (B) Schematic diagram of the integrated digital microfluidic platform using electromagnet for influenza A (infA)/H1N1 detection. Reproduced with permission from Lu et al. [99]. Copyright 2020, Royal Society of Chemistry. (C) Schematic view of the hybrid-magnet-based magnetic microbead manipulation device and the layout of the array of planar coils (i). Two permanent magnets (ii) were symmetrically placed on a soft magnetic sheet (iii) to generate a vertical uniform magnetic field B_0_. Reproduced with permission from Rida et al. [101]. Copyright 2003, American Institute of Physics. (D) Schematic illustration of the magnetic bead (MB)−quantum dot (QD) assay based on the hybrid magnet system. PDMS, polydimethylsiloxane; PL, photoluminescence. Reproduced with permission from Kim et al. [104]. Copyright 2017, American Chemical Society.

In magnetic droplet systems, splitting magnetic beads from mother droplet is the key to realize multiple immunoassays. Efficient adsorption of biomolecules often requires a considerable number of magnetic particles, which can impede droplet control due to the small ratio of liquid to particles, thereby making it difficult to split the magnetic beads from the droplet. Zhang et al. [76] incorporated a surface topographic feature on the surface to facilitate the droplet manipulation. As shown in Fig. 6A, the slits formed by the microsurface elevation can provide high surface tension and friction barriers for the splitting of magnetic bead from droplets. Sample-to-answer detection of the cancer biomarker remodeling and spacing factor 1 and *E. coli* was achieved combining with real-time polymerase chain reaction (PCR) and real-time helicase-dependent amplification. Besides, the transfer of magnetic beads between 2 chambers that contain droplets may induce surface deformation at the boundaries between the water-based reagent and oil. To eliminate this phenomenon, Kim et al. [91] developed a microfluidic chip for magnetic bead droplet manipulation using micropillars between 2 adjacent chambers to form stable water–oil interfaces to reduce the magnetic bead loss during the transfer between 2 chambers. Results showed that the liquids were clearly and stably separated in each chamber by the designed micropillars and a sensitive detection of oligomer Aβ with a limit of detection of 20 pg/ml was achieved in the serum-spiked sample.

Magnetic manipulation of droplets using permanent magnets has gained popularity as a power-free solution for long-term outdoor biochemical analysis, and it can be conveniently accomplished through translational systems. In addition, the magnetic force from permanent magnet onto magnetic beads is strong enough for transferring magnetic beads between droplets, which could effectively reduce the cost of the magnetic beads. However, it should be noted that the lateral dimension of the permanent magnets utilized in these systems is typically in the millimeter scale, which can limit the resolution of droplet manipulation. Furthermore, such manipulation typically lacks strategies for magnetic bead agitation or dispersion, as the permanent magnet alone cannot adjust the magnetic field strength or polarity, leading to a potentially reduction in immune reaction and washing efficiency.

#### Electromagnet-based droplet manipulation

Electromagnets represent a more flexible approach for magnetic droplet manipulation when compared to permanent magnets, owing to their ability to occupy smaller spaces and assume diverse geometries. Besides, electromagnets can effectively modulate the strength of the magnetic field by varying the current flowing through them.

Different from the single-magnetic-bead control presented in some early work [92–94], manipulation of magnetic bead-contained droplet using magnetic force required a stronger magnetic field, which needs relatively high current density for electromagnets. The major issue with the increasing current in electromagnets is joule heating. To mitigate heating and enhance the intensity of the electromagnetic field, researchers have explored optimizing the geometrical parameters of microelectromagnets or adding a ferromagnetic core to achieve the maximum magnetic force. One typical structure of microelectromagnet used in magnetic droplet is magnetic tweezer/needle [95–98], which is consisted of an iron core with sharp tip and assembled coil around iron core. In magnetic-tweezer-based magnetic manipulation systems, it is important to adjust the balance between magnetic force, surface tension, and hydrodynamic force. To achieve and simplify this balance, the droplets were often confined at a continuous oil flow in a channel, and the magnetic tweezer was fixed and placed near the channel, so that the hydrodynamic forces on the main droplet exceed the 2 other forces. This simplification allows for easier analysis of the system and accurate determination of the relative contributions of each force, as the balance of magnetic force and surface tension should only be evaluated. The activated magnetic tweezer could form high intensity and gradient magnetic field around the sharp tip and trap the magnetic beads from droplets. Lu et al. [99] presented a magnetic digital microfluidic platform using tunable electromagnets focused on a structure-free, superhydrophobic surface (Fig. 6B). The magnetic bead-contained droplet could be manipulated on the hydrophobic surface automatically. Yang et al. [100] proposed a modified electromagnet needle integrated with a superhydrophobic coating for magnetic droplet manipulation on an open surface. The system provided a simple, cost-effective but powerful platform for droplet manipulation, including droplet transport, fusion, mixing, and magnetic beads extraction, in a 3D mode. The superhydrophobic coating on the modified electromagnet needle allowed direct contact with droplets, without wetting, or contamination problems.

#### Hybrid-magnet-based droplet manipulation

The previous sections have shown that both permanent magnets and electromagnets are suitable for the manipulation of magnetic droplets in biological applications. The properties of the magnetic fields generated by the 2 types of magnet systems are strongly complementary. The static magnetic field generated by the permanent magnet can provide a basic magnetic field with high intensity, while the electromagnets can produce an adjustable magnetic field with high gradient. By combining electromagnets and permanent magnets into a hybrid magnet system, 2 main advantages can then be obtained: (a) The basic static magnetic field provided by the permanent magnet can result in a high intensity; thus, less current for the electromagnetic field is required, and the joule heating effect can be well reduced. (b) The hybrid magnet system allows for the precise adjustment of the local magnetic field, which is more flexible for the manipulation of magnetic droplets than the permanent magnet one.

One typical work using 2 bar-shaped NdFeB (40 × 15 × 8 mm^3^) permanent magnets combined with a printed circuit board to develop a hybrid magnet system was proposed by Rida et al. [101]. In this work, 2 permanent magnets were placed on the top of a soft magnetic sheet to generate a basic uniform static magnetic field with the intensity of 550 mT along the entire length of a microfluidic glass capillary (as shown in Fig. 6C). Under the assistant with the basic static magnetic field, energized printed circuit board microcoils (400 A/mm^2^) can help to generate a magnetic field with a local maximum gradient of 5 mT/mm. The manipulation of the magnetic particles between droplets can be achieved by the local magnetic field with high gradient. Some optimizations of the pattern of the microcoils were demonstrated to provide more efficient magnetic droplet operation with larger range [102,103]. The physical structures of the microfluidic channel have also been presented to help the manipulation of magnetic droplet in hybrid magnet system. Chiou et al. [77] developed a sieve structure on an open surface to assist droplet splitting operation by the coil array-induced magnetic field gradient and integrated the PCR-based genetic testing in the electromagnetic droplet platform. The utilization of sieve structures with small gap can assist droplet deformation by alleviating the effective capillary force; thus, a relatively low magnetic force was only required for the magnetic beads transfer. Following a similar principle, Kim et al. [104] used a closed microchannel with low height connected to 2 chambers containing droplets to assist the droplet manipulation (Fig. 6D). In addition, the immunoassay for the detection of histidine-rich protein 2 was successfully developed on the basis of the capture and quantum dots using magnetic beads.

### Magnetic bead in microwell

The precise and sensitive detection of the biomolecules is an essential aspect of disease diagnosis and therapeutic monitoring. The present laboratory techniques, such as enzyme-linked immunosorbent assay and PCR, have been widely utilized for the sensitive detection of the biomarkers in patient samples. However, the concentrations of several crucial biomarkers in patient samples often fall below the detection limits of these conventional laboratory methods. Recent developments in the field of bioanalytical technologies have enabled ultrasensitive analysis of biologically relevant molecules, especially those with low concentrations. Among these technologies, magnetic-bead-based digital bioassays have gained more attention, as they allow for the isolation and digital detection of single molecules. By capturing single target analytes in a microwell array using magnetic beads, these bioassays can reach to an attomolar detection sensitivity. In such bioassays, the target were first captured by the magnetic beads preisolated in the microwell with single-bead resolution, and then fluorescent signal was generated and detected on the basis of fluorescent amplification reaction. Thus, the efficient seeding of magnetic beads into microwells is critically important.

The first examples of microwell arrays were prepared using optical fiber bundles exposed in an acid bath [105]. On the basis of these fiber optical microwells, a bead array platform was developed by Illumina to achieve high-throughput nucleic acid analysis [106]. Although this platform can achieve a high bead retention rate of >97% by the evaporation-assisted seeding method, the fabrication process using optical fiber glass bundles is costly, and aggressive chemicals are required. Besides, the evaporation-assisted seeding process may cause protein denaturation and the loss of enzyme activity, which limits its biological application. Currently, microwells are commonly prepared by microfabrication such as photolithography [107,108], electrochemical micromachining [109,110], and microcontact printing [111,112]. To improve the loading efficiency of the magnetic beads, microwells with a hydrophilic bottom and hydrophobic interwell surface have been manufactured [113,114]. The hydrophilic bottom could help to trap the magnetic beads in aqueous solution into the microwell, while the hydrophobic surface could better adhere the oil to avoid the escape of beads. Besides, placing magnets underneath the microwell substrate could also attract the magnetic beads by magnetic force and trap the beads into microwell to enhance the loading efficiency [115–117].

The main advantage of this technology is its ability to detect single molecules with high sensitivity and specificity. Besides, it can be used to analyze multiple targets simultaneously using different types of magnetic beads functionalized with different biological recognition elements. On the basis of the microwell arrays, many recent researches were presented for single-biomolecule detection such as proteins [118,119], nucleic acids [120,121], and cells [122,123]. Safdar et al. [116] demonstrated the multiplex detection of nucleic acid targets using magnetic beads in femtoliter-sized microwells. In this work, ultrasensitive detection of nucleic acid with a lower detection of 180 fM was achieved without any target amplification or signal amplification strategies. Rissin et al. [118] proposed a method for the protein detection in blood using microwells with a volume of 50 fl. The digital enzyme-linked immunosorbent assay was performed in such microwells by loading magnetic bead with a diameter of 2.7 μm and can detect PSA with the limit of detection of 50 aM.

## Conclusions and Future Prospects

In recent years, there has been a growing interest in the application of the magnetic beads for capturing and detecting different compounds. With the development of microfluidics, magnetic-based biological applications have been successfully miniaturized and integrated into microfluidic systems. In this paper, recent advances in magnetic manipulation methods for magnetic beads in microfluidic system have been reviewed. The advances in microfluidics and magnetic bead manipulation have resulted in a remarkable progress in various biological applications, as demonstrated by the notable works listed in Table. The main role of magnetic beads in these works was actually a carrier of biological recognition molecules for capturing specific biomarkers. Actually, magnetic bead can also be used as a signal output itself. There are several advantages directly using magnetic beads as detection signal in biological detection: (a) low cost. Only one biological recognition molecule is required, and no addition labeling material is required. (b) Rapid detection time. Compared with the conventional immune-based methods, which required 2 kinds of antibodies for reaction recognition, methods that directly use magnetic beads as both target capture carrier and signal carrier can greatly shorten the detection time by only one step of immune reaction. (c) Simple procedure. Magnetic-bead-based assay is easy to operate without laborious pretreatment and purification as most biological and environmental samples intrinsically have a low magnetic background.

**Table.** Biological applications using magnetic bead manipulation in microfluidic chip.

Applications		Methods	Throughput	Separation efficiency	Limit of detection	Reference
**Cell sorting**	Red blood cell	Magnetophoresis	5 μl/h	91.1%	/	[129]
	White Blood cell	Magnetophoresis	15 μl/h	100%	/	[130]
	Stem cell	Magnetophoresis	1 × 10^7^ cells per test	92.1%	/	[131]
	Cancer cell	Magnetophoresis	1,000 μl/min	92.5%	/	[132]
	Bacteria	Magnetic bead chains	1 ml/min	85%	10^2^ CFU/ml for *E. coli*	[57]
		Magnetophoresis	40 ml/h	>96%	/	[133]
		Magnetic fluidized bed	1 μl/min	/	4 CFU/ml for *Salmonella* and *E. coli*	[72]
**Nucleic acid assay**	Nucleic acid separation and extraction	Magnetophoresis	40 μl/h	98.4%	/	[134]
		Magnetophoresis	0.01-0.05 m/s	>80%	/	[43]
	Nucleic acid detection	Magnetic fluidized bed	5 μl/min	93%	0.1 fM for padlock probes	[71]
		Magnetic bead in microwell	25 μl per test	/	180 fM for nucleic acid	[116]
		Magnetic bead in microwell	/	80%	1 × 10^2^ copies/ml for influenza virus	[121]
**Immunoassay**	Electrochemical immunoassay	Magnetic bead chains	150 μl/min	51%	10^1^ CFU/ml for *Salmonella*	[54]
		Magnetic bead chains	10 μl/test	/	10 mIU/ml for β-human chorionic gonadotropin	[135]
		Magnetic bead chains	0.15 ml/min	50%	10^1^ CFU/ml for *Salmonella*	[136]
	Optical immunoassay	Magnetic droplet	5 samples per test	/	0.044 ng/ml for procalcitonin	[89]
		Magnetic droplet	/	/	0.032 hemagglutination unit for H1N1	[99]
		Magnetic bead chains	/	95% for PSA; 90% for AFP; 91% for CEA;	10 pg/ml for PSA, AFP, and CEA	[65]
		Magnetic bead in microwell	/	/	0.4 fM for PSA	[118]
	Direct magnetic immunoassay	Magnetic relaxation switching	800 μl per test	/	10^2^ CFU/ml for *Salmonella enterica*	[127]
		Aggregation of immunomagnetic beads	900 μl per test	/	10^4^ CFU/ml for *E. coli*	[128]

So far, many researches have demonstrated using magnetic beads as signal output in biological detection, which are also summarized in Table. The typical method is using magnetic relaxation switching, which is based on the aggregation or disaggregation of magnetic beads induced by the target [124–126]. In this method, the transverse relaxation time (*T*_2_) of surrounding water protons will be changed when the superparamagnetic nanobeads are aggregated around target molecules due to the external magnetic field. The change of the *T*_2_ (Δ*T*_2_) is related to the amount of target present in the sample. As a result, the magnetic beads can be directly utilized as the signal output in such assays without any other signal probe molecules, such as quantum dots, enzymes, etc. Chen et al. [127] presented a one-step approach for detecting pathogens and viruses through magnetic relaxation switching method. Magnetic beads with 2 different sizes (MB_250_ with a diameter of 250 nm and MB_30_ with a diameter of 30 nm) were used in their work to offer different separation speeds in a magnetic field with low intensity (0.01 T). Because of the larger size of MB_250_, it experienced a greater magnetic force under the same magnetic field conditions than MB_30_. The MB_250_ can be rapidly separated by the magnetic field within 1 min due to the large magnetic force it experienced, while the MB_30_, used as the magnetic probe, remains unseparated even after 60 min. Thus, the Δ*T*_2_ of the water molecules surrounding the unreacted MB_30_ is chosen as the readout of the immunoassay. Another interesting method based on the optical image signal of the aggregated magnetic beads for biological detection was also proposed by the same group [128]. In their work, the biotarget were first reacted with immune magnetic beads in a tube, and then one permanent magnet was placed next to the tube for the magnetic separation. The magnetic beads can be specifically bound to targets and attracted and agglomerated on the side of the tube to form a narrow brown strip due to large magnetic force, while the free magnetic beads will form a dispersed wide strip. The width and color of the strip can be used for quantitative detection.

Although the magnetic manipulation system has been greatly developed, it still has many remaining challenges when facing industrial and clinical applications:

1. The current magnetron microfluidic chips still have a low sample processing throughput, which cannot meet the needs of large-scale clinical testing. Therefore, one potential future direction is to increase the number of magnetron sources on the chip to enable simultaneous processing of multiple samples, which can effectively improve the sample processing throughput without sacrificing the high sensitivity and specificity of the detection. Another future direction is to integrate novel nanotechnologies into microfluidic chips, which can enable the development of more sophisticated and powerful microfluidic systems with higher throughput and sensitivity. Besides, how to improve the existing integrated electromagnetic coil processing technology, make micromagnets with higher magnetic field gradient and strength, and strengthen the process standardization and system stability are also of great significance for the future industrial development.
2. A fully automated magnetic manipulation platform is still urgently required as the current systems mainly rely on open-loop control algorithms and could only perform the magnetic control by a given set of predetermined parameters. Closed-loop feedback based on image recognition can provide a noncontact signal acquisition method, which is suitable for magnetic manipulation system. The key challenge to achieve image-based feedback in magnetic manipulation system is how to quickly identify droplets and magnetic particles. As the edges of the droplets are normally transparent, using traditional target recognition methods based on Hough transform and edge detection algorithms is time-consuming and inefficient. The neural network generated by targeted training can well identify the obscure targets. Thus, combining with the artificial intelligence has the potential to generate a rapid closed-loop feedback system for monitoring manipulation in real time and guide the system to rectify the problems before moving on to the next step.
3. Magnetic beads with different sizes will experience different magnetic forces, which will directly affect the stability and repeatability of the magnetic system, especially in magnetophoresis and magnetic droplet system. Thus, the size uniformity of the magnetic beads used in the microfluidic system is also a critical issue. One approach to achieving greater uniformity could be to improve the manufacturing processes used to produce the beads. This might involve developing more precise techniques for controlling the size and shape of the beads or introducing new materials or technologies to improve their consistency. Another approach is developing innovative particle separation techniques to achieve accurate sorting of magnetic beads with given size.
